# Supplementary material for: Distinct responses of COL17A1 nonsense variants to readthrough drugs and NOG as a novel enhancer in junctional epidermolysis bullosa
Source: Mol Ther Nucleic Acids. 2026 Sep 3;37(4):103088. doi: 10.1016/j.omtn.2026.103088 (PMC13628650; doi:10.1016/j.omtn.2026.103088)
Supplement: Supplementary file 1 [file mmc1.pdf]

OMTN, Volume *37*

Supplemental information

Distinct responses of *COL17A1* nonsense variants to readthrough drugs and NOG as a novel enhancer in junctional epidermolysis bullosa

Yan Tan, Haifa El Mabrouk, Rui Zhang, Sato Magassa, Cristina Has, and Saliha Beyza Sayar

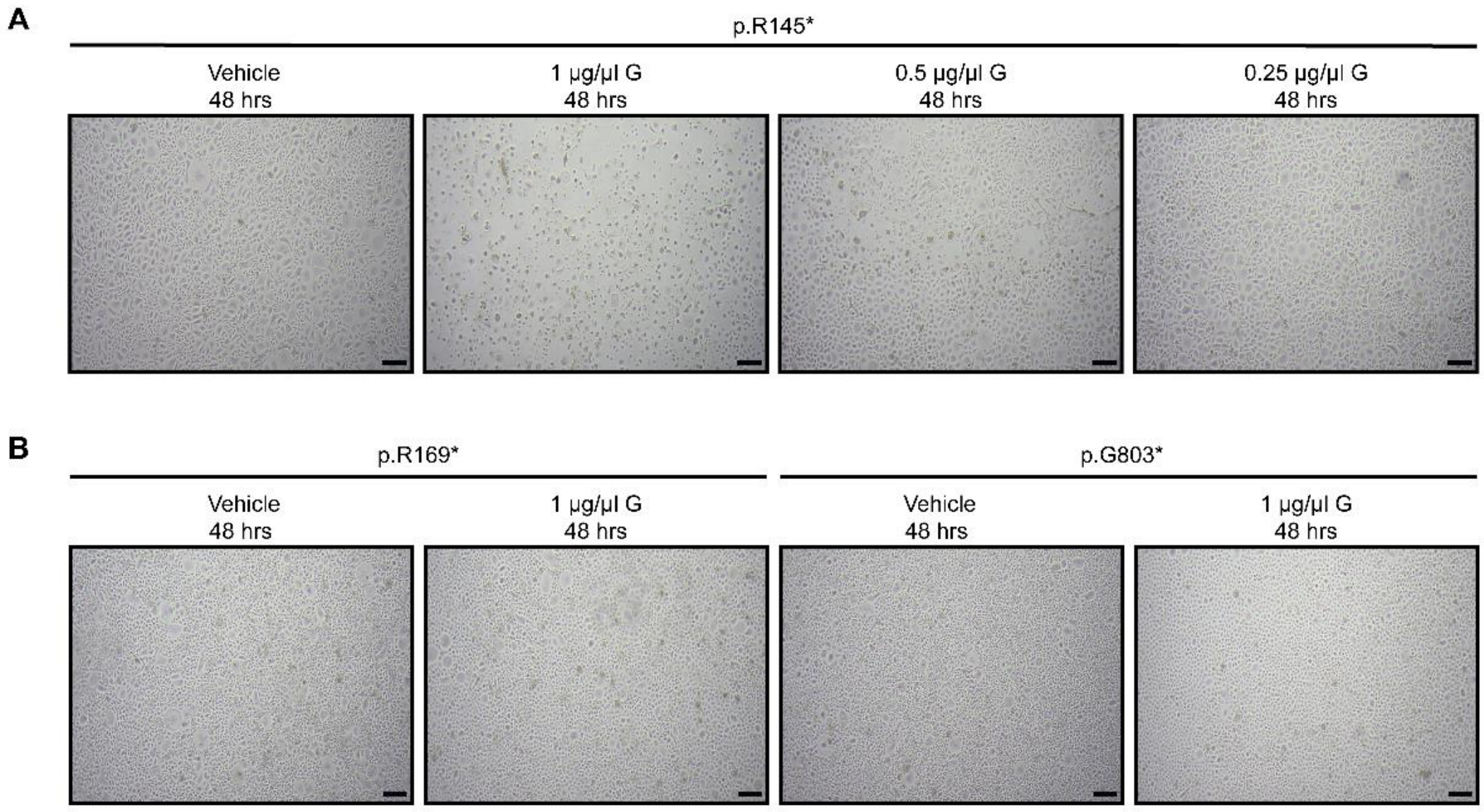

Figure S1. Morphology of JEB-C17 keratinocytes with high dosage G treatment.

(**A**) Morphological changes of JEB-R145* keratinocytes treated with G (1 µg/ µL, 0.5 µg/ µL, or 0.25 µg/ µL) respectively. (**B**) JEB-R169* and -G803* cells maintained normal morphology following treatment with G (1 µg/ µL). Cells were treated for two consecutive days and the pictures were taken at 48 hrs. Vehicle treated cells were used as reference controls. G, gentamicin. Bar = 50 µm.

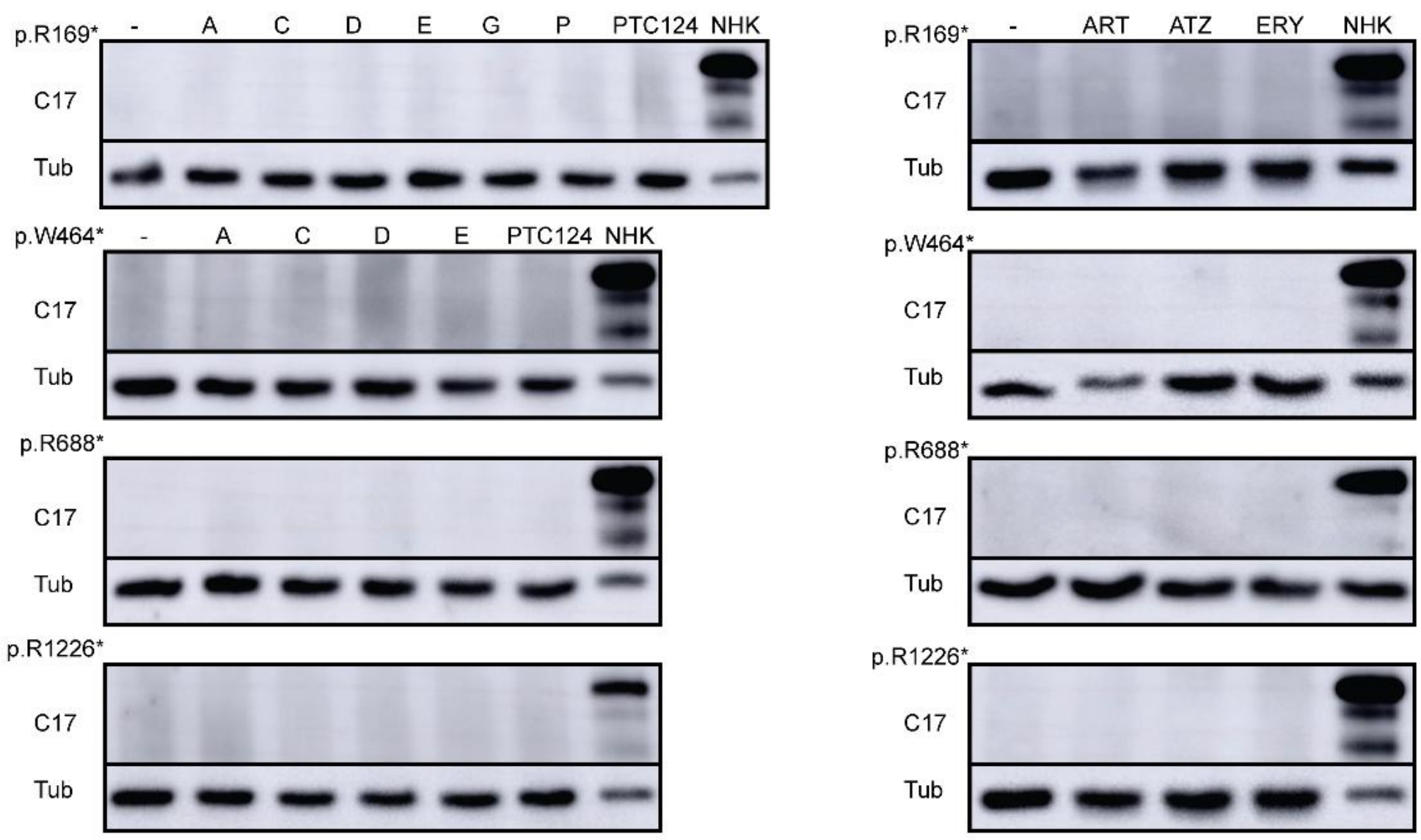

Figure S2. Screening of TRIDs in JEB-C17 keratinocytes.

Immunoblot analysis of C17 in JEB-R169*, -W464*, -R688* and -R1226* following treatment with TRIDs (A 25 µM, ART 32 µM, ATZ 6.4 nM, C 2.7 µM, DAP 100 µM, E 3.5 µM, ERY 8 µM, G 1µg/µL, P 1µg/µL, and PTC124 35 µM) versus vehicle. Cells were treated for two consecutive days. Fourfold higher input was used for JEB-C17 keratinocytes as compared to NHKs. Tubulin served as a loading control. NHKs were used as reference controls; vehicle treated cells are indicated by "–". Data represent n = 2. A, amlexanox; ART, artesunate; ATZ, atazanavir; C, clitocine; D, 2,6-diaminopurine; E, ELX-02; ERY, erythromycin; G, gentamicin; P, paromomycin; PTC124, ataluren.

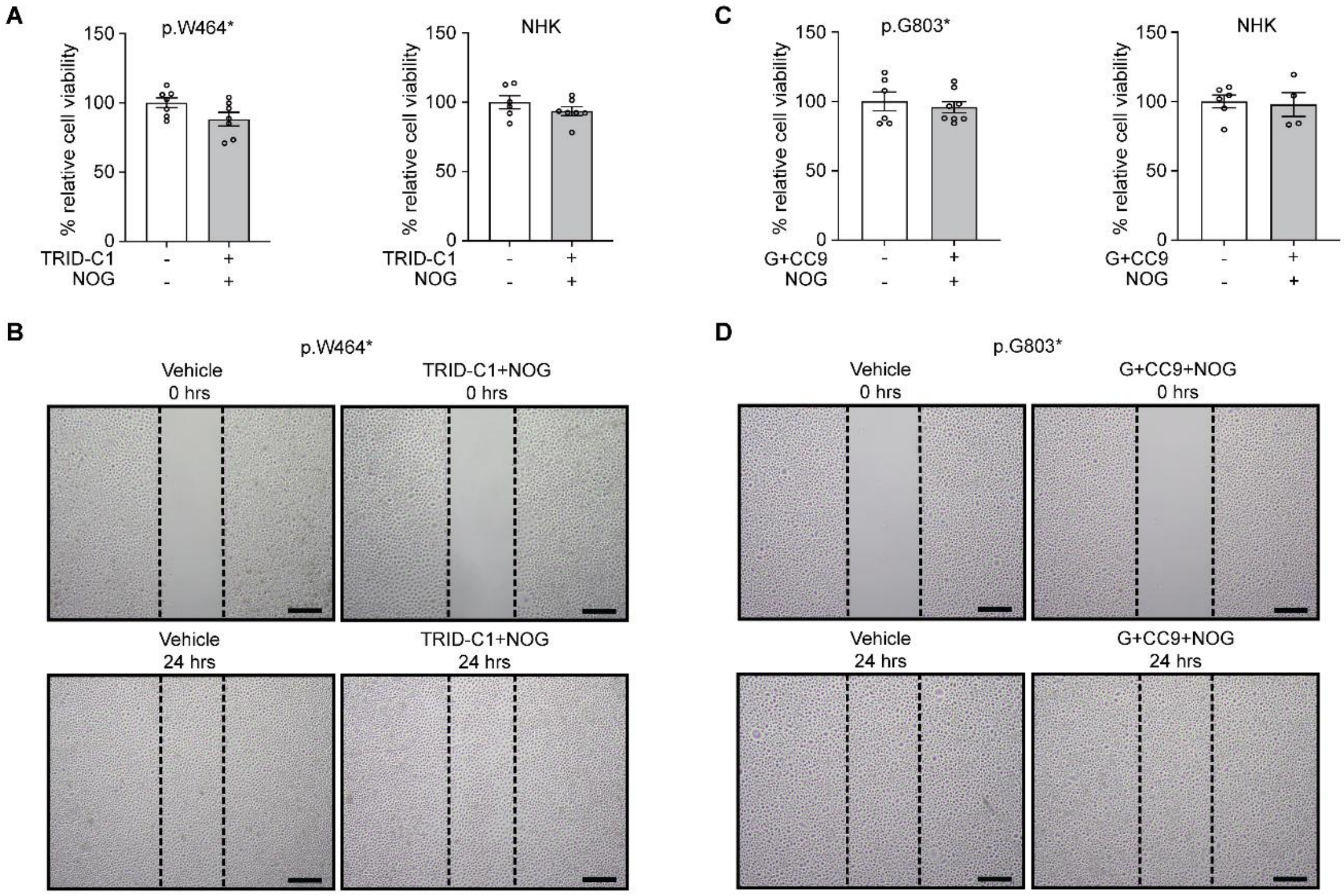

Figure S3. Evaluation of NOG effect on cell viability and *in vitro* wound closure.

(**A**) The graph depicts the relative change of cell viability of JEB-W464* and NHKs treated with NOG 5 µM in combination with TRID-C1 (G 0.003 µg/µL, P 0.05 µg/µL, and CC-90009 30 nM). (**B**) *in vitro* wound closure assay of JEB-W464* and NHKs treated with NOG 5 µM in combination with TRID-C1. (**C**) The graph shows the relative change of cell viability of JEB- JEB-G803* and NHKs treated with NOG 5 µM in combination with G 0.003 µg/µL and CC-90009 30 nM. (**D**) *in vitro* wound closure assay of JEB-G803* and NHKs treated with NOG 5 µM in combination with G 0.003 µg/µL and CC-90009 30 nM. Bars = 50 µm. Data are presented as mean ± SEM (**P* < 0.05, ***P* < 0.01, ****P* < 0.001; n = 3). Vehicle treated cells are indicated by "–". CC9, CC-90009.

Table S1. Primers used for RT-qPCR

Primer Name	Direction	Sequence 5′−3′	Annealing Temperature (°C)	Product Size (bp)
*COL17A1*_ex40-44	F	GAAACCTTCCTCTCCGGCC	60	232
*COL17A1*_ex40-44	R	CTAGGAATGCCAAGAGCCCC	60	232
18S	F	GTAACCCGTTGAACCCCATT	60	151
18S	R	CCATCCAATCGGTAGTAGCG	60	151
TBP	F	CATTGGACTAAGATAGGGATTCCG	60	93
TBP	R	ATGGATCAGAACAACAGCCTGC	60	93

Legend: F, forward; R, reverse.
