# Supplementary material for: Distinct responses of COL17A1 nonsense variants to readthrough drugs and NOG as a novel enhancer in junctional epidermolysis bullosa
Source: Mol Ther Nucleic Acids. 2026 Sep 3;37(4):103088. doi: 10.1016/j.omtn.2026.103088 (PMC13628650; doi:10.1016/j.omtn.2026.103088)
Supplement: Supplementary file 2 [file mmc2.pdf]

Molecular Therapy
Nucleic Acids
Original Article

Distinct responses of *COL17A1* nonsense variants to readthrough drugs and NOG as a novel enhancer in junctional epidermolysis bullosa

Yan Tan,[1] Haifa El Mabrouk,[1] Rui Zhang,[1] Sato Magassa,[1] Cristina Has,[1] and Saliha Beyza Sayar[1]

[1]Department of Dermatology, Medical Center – University of Freiburg, 79104 Freiburg, Germany

Junctional epidermolysis bullosa (JEB) caused by *COL17A1* pathogenic variants (JEB-C17) is characterized by skin fragility, chronic wounds, and an increased risk of squamous cell carcinoma. Approximately 20% of cases are caused by nonsense mutations, which induce premature termination codons (PTCs) resulting in truncated protein production and/or trigger mRNA degradation through nonsense-mediated decay (NMD) pathway. Different approaches have been suggested for nonsense mutation suppression including translational readthrough- inducing drugs (TRIDs) therapy, which is extensively examined in hereditary skin diseases due to relatively low costs for translation into the clinic. We evaluated a panel of TRIDs in patient-derived JEB-C17 keratinocytes carrying different *COL17A1* nonsense variants. We observed variable responses to TRIDs across mutations. N-oxalylglycine (NOG) has been shown to reduce eRF1 hydroxylation and consequently inhibit NMD. We investigated NOG as an enhancer of aminoglycoside-mediated readthrough for JEB-C17. We previously developed a cocktail consisting of aminoglycosides, NMD inhibitors as enhancers of readthrough activity, and antioxidants that act as anti-inflammatory agents. In this study, we explored the effect of NOG as a readthrough enhancer in combination with TRIDs or TRID-cocktail. Our results support mutation-dependent TRID selection for further preclinical scientific investigations and highlight personalized therapeutic approaches as a key strategy for JEB-C17.

INTRODUCTION

Junctional epidermolysis bullosa (JEB) caused by *COL17A1* pathogenic variants (JEB-C17) is primarily characterized by skin blistering, leading to wounds that may become chronic and progress to squamous cell carcinomas, which are life-threatening.[1] Current management remains largely symptomatic, focusing on wound care and supportive measures, while effective curative therapies are still lacking.[2] Although *in vivo* gene therapies have been approved for dystrophic epidermolysis bullosa, comparable approaches have not yet been developed for JEB, partly due to its ultra-rare nature and limited interest from pharmaceutical companies.[2,3] Prime editing and other RNA or DNA editing strategies are in preclinical development.[4,5]

Approximately 20% of JEB-C17[6,7] cases are caused by nonsense mutations which induce premature termination codons (PTCs) resulting in truncated protein production (often nonfunctional or partially functional) and/or trigger mRNA degradation through the surveillance pathway nonsense-mediated decay (NMD) which prevents harmful truncated protein production.[8–10] Translational readthrough-inducing drug (TRID) therapy is one of the approaches that has been suggested for a nonsense mutation suppression therapy alongside tRNA suppressor, RNA editing, antisense-oligos, and CRISPR technology.[11–13] Due to relatively low costs for translation into the clinic, TRID therapy is extensively examined in hereditary skin diseases.[14]

We have showed that aminoglycosides induced readthrough activity on JEB-W464* and induce restoration of less than 1% of collagen XVII (C17) relative to normal human keratinocytes (NHKs), only at high concentrations with significant offtarget effects on gene expression.[7] In our previous work, we developed an aminoglycosides-based cocktail (TRID-C5). It consists of low doses of gentamicin (G) and paromomycin (P) with NMD inhibitors, CC-90009, NMDI-14, to enhance the readthrough activity, and the antioxidants, apocynin, melatonin, act as anti-inflammatory agents.[15] TRID-C5 induced C17 readthrough in both primary and immortalized JEB-W464* keratinocytes,[15] indicating that observed effects were comparable in primary and immortalized cells.

In this study, we evaluated a panel of TRIDs across six *COL17A1* nonsense variants to select effective compound for TRID cocktail development. We used patient-derived immortalized keratinocytes, which provided a stable and disease-relevant experimental model.[16] Amlexanox,[17] ataluren (PTC124),[18] G, P,[19–22] ELX-02,[23] clitocine,[13] artesunate, atazanavir, and erythromycin[24–26] that had already been shown to promote readthrough activity in other subtype of JEB.

Received 5 June 2026; accepted 2 September 2026;
https://doi.org/10.1016/j.omtn.2026.103088.

Correspondence: Cristina Has, Department of Dermatology, Medical Center – University of Freiburg, 79104 Freiburg, Germany.
E-mail: cristina.has@uniklinik-freiburg.de
Correspondence: Saliha Beyza Sayar, Department of Dermatology, Medical Center – University of Freiburg, 79104 Freiburg, Germany.
E-mail: saliha.beyza.sayar@uniklinik-freiburg.de

Published by Elsevier Inc. on behalf of The American Society of Gene and Cell Therapy.
This is an open access article under the CC BY license (http://creativecommons.org/licenses/by/4.0/).

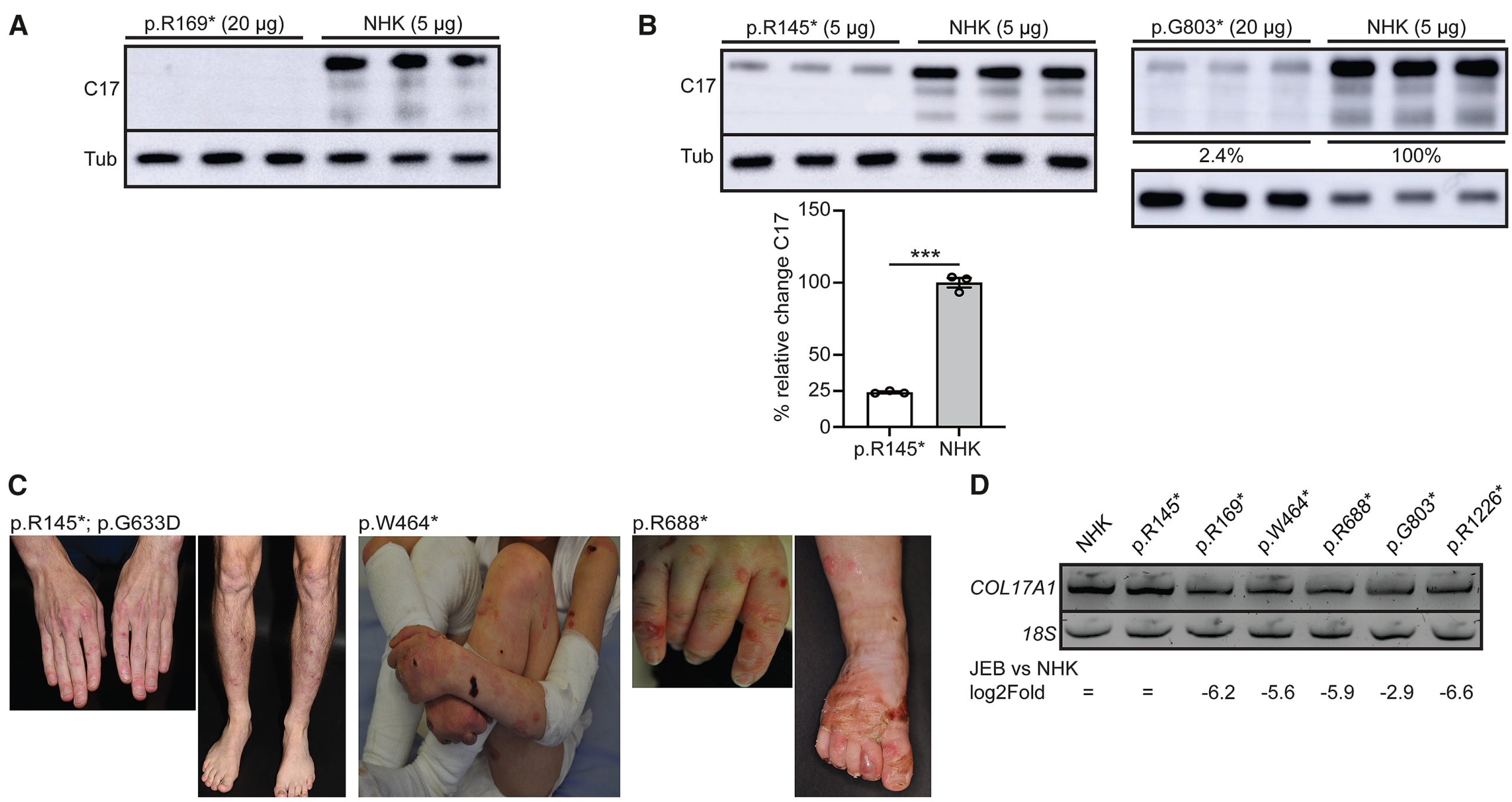

Figure 1. Genotype-phenotype correlation in JEB-C17

(A and B) Immunoblot analysis of C17 in JEB-R145* and -G803*, compared with NHKs. Equal protein amounts were loaded for JEB-R145*, while 4-fold higher protein input was used for JEB-R169* and -G803*. Tubulin served as a loading control. Data are presented as mean ± SEM (*$p < 0.05$, **$p < 0.01$, ***$p < 0.001$; $n = 3$). (C) Representative clinical images of patients corresponding to the indicated genotypes. (D) *COL17A1* mRNA expression in JEB- R145*, -R169*, -W464*, -R688*, -G803*, and -R1226*, compared with NHKs, analyzed by RT-PCR and RNA-seq. Data represent $n = 3$. Tub, tubulin.

2,6-diaminopurine[27] and clitocine[28,29] previously demonstrated efficacy in other nonsense mutation models such as cystic fibrosis, Duchenne muscular dystrophy, and cancer. Moreover, N-oxalylglycine (NOG) has been reported to inhibit NMD via reduction of eRF1 hydroxylation in a cancer reporter cell line.[30,31] We explored the effect of NOG as a readthrough enhancer in combination with TRIDs or TRID-cocktail. The aims of our study are to determine the most effective TRID(s) and to optimize the TRID-cocktail, thereby providing the preclinical scientific basis for development of the therapies for JEB-C17.

RESULTS

Characterization of patient-derived JEB-C17 keratinocytes

C17 was undetectable in keratinocytes carrying the heterozygous nonsense mutation p.R169* (Figure 1A), as well as in those carrying the homozygous nonsense mutations p.W464*, p.R688*, and p.R1226*, as shown previously.[7] In contrast, JEB-R145* and JEB-G803* retained approximately 24% and 2.4% of normal C17 levels, respectively, due to compound heterozygosity with splice site mutations (Figure 1B; Table 1). Due to these differences, direct comparison with NHKs required adjusted protein loading in immunoblot. The amount of C17 appeared to correlate with the clinical severity observed in the patients (Figure 1C; Table 1). *COL17A1* mRNA was detected in all cell lines but showed variable reduction, with decreased transcript levels in JEB-R169* ($\log_2$FC = −6.2), -W464* (−5.6), -R688* (−5.9), -G803* (−2.9), and -R1226* (−6.6), whereas in JEB-R145*, *COL17A1* mRNA levels were comparable to NHKs (Figure 1D), indicating variability in NMD.

TRID screening in JEB-C17 keratinocytes

We evaluated a panel of TRIDs, which are amlexanox, artesunate, atazanavir, clitocine, 2,6-diaminopurine, ELX-02, erythromycin, G, P, and PTC124 in the above characterized JEB-C17 keratinocytes (Table 1). High concentrations of G (1 μg/μL) and P (1 μg/μL) were initially used for screening to maximize the readthrough effect.[7] Cells were treated with individual compounds for two consecutive days and harvested at 48 h. Following treatment with G at concentrations of 1 and 0.5 μg/μL, JEB-R145* keratinocytes became rounded and detached from the culture dish, rendering them unsuitable for downstream immunoblot analysis (Figure S1A). Therefore, a lower concentration of G 0.25 μg/μL was selected for subsequent experiments. In contrast, JEB-R169* or -G803* keratinocytes maintained normal morphology following treatment with G at 1 μg/μL (Figure S1B). Furthermore, PTC124 (35 μM) also induced readthrough activity in JEB-R145* keratinocytes (Figure 2A).

JEB-W464* showed robust responses to high doses aminoglycosides, whereas JEB-R688* exhibited a weak response but JEB-R1226* did not respond (not shown here; see[7]). The other compounds did not induce noticeably readthrough in JEB-R145*, -W464*, -R688*,

Table 1. Characterization of patient -derived JEB-C17 keratinocytes

Nonsense mutations					
cDNA level	Protein level	Second mutations	C17 amount[a]	Phenotype of the patient	Reference
c.433C>T	p.R145*	c.1898G>A; p.G633D	24%	localized blistering	Kiritsi et al.[32]
c.505C>T	p.R169*	c.4156 + 1G>A; SS	negative	localized blistering	Kiritsi et al.[32]
c.1392G>A	p.W464*	homozygous	negative	generalized blistering	Kiritsi et al.[32]
c.2062C>T	p.R688*	homozygous	negative	generalized blistering	Condrat et al.[6]
c.2407G>T	p.G803*	c.3198C>T; p.S1066S	2.4%	localized blistering	Hoffmann et al.[33]
c.3676C>T	p.R1226*	homozygous	negative	generalized blistering	Kiritsi et al.[32]

Legend: [a], relative to NHKs in immunoblot analysis.

-G803*; or -R1226*, in two experimental replicates. In addition, JEB-R169* did not exhibit detectable readthrough in response to any tested TRIDs (Figure S2). Together, these findings demonstrate variability in readthrough responses across nonsense mutations.

Based on the screening results, G and PTC124 were selected for further evaluation. To improve the translational potential of aminoglycoside-based therapy, different concentrations of G were combined with NMD inhibitors and/or antioxidants. The resulting combinations (TRID-cocktails) were subsequently evaluated in JEB-R145* and JEB-G803* keratinocytes, whereas JEB-W464* served as a positive control based on our previous demonstration of TRID-C5 (a G- and P-based cocktail)-mediated readthrough.[15]

NOG enhances G and P-mediated readthrough

We next evaluated NOG as a readthrough enhancer in combination with a cocktail containing G, P, and CC-90009 (designated as TRID-C1). In JEB-W464*, addition of NOG increased C17 levels in a dose-dependent manner, with no significant difference between 5 and 10 μM. The addition of 5 μM NOG increased C17 levels by approximately 70% compared with TRID-C1 alone (Figure 3A), without affecting cell viability or *in vitro* wound closure capacity (Figures S3A and S3B). Combination of NOG 5 μM with TRID-C1 restored approximately 15% C17 in JEB-W464* relative to NHKs (Figure 3B). Furthermore, the addition of melatonin (TRID-C2) or melatonin and apocynin (TRID-C3), and NMDI-14 (TRID-C5) to NOG-TRID-C1 did not further increase C17 levels, and no significant difference was observed between NOG+TRID-C1 and TRID-C5 (Figure 3C).

At the transcript level, NOG in combination with TRID-C1 increased *COL17A1* mRNA levels by approximately 2.7-fold, whereas NOG or CC-90009 alone or their combination had no significant effect (Figure 3D, left), indicating that the effect of TRIDs is the initial step for readthrough activity. In JEB-R1226*, NOG or CC-90009, alone or in combination with TRIDs (G and P) did not increase *COL17A1* mRNA levels (Figure 3D, right).

In cells that only responded to G, such as JEB-G803*, 5 μM NOG increased C17 levels by approximately 60% in combination with G and CC-90009 (Figure 3E) without affecting cell viability or *in vitro* wound closure capacity (Figures S3C and S3D) whereas no enhancement was observed in JEB-R145* (Figure 3F). Furthermore, NOG did not enhance PTC124-mediated readthrough in JEB-R145* (Figure 3G).

G-based TRID-cocktails

Given that JEB-R145* and -G803* responded to G, we further evaluated G-based TRID-cocktails. Low dose G 0.003 μg/μL combined with CC-90009 30 nM, NMDI-14 100 nM, apocynin 25 μM, and melatonin 25 μM increased C17 levels by 38% in JEB-G803* (Figure 4A) but had no effect in JEB-R145* (Figure 4B). Increasing G concentrations did not further enhance C17 levels in neither JEB-G803* or -R145*. Due to low baseline expression, 4-fold higher protein input was required for JEB-G803* samples compared with NHKs but absolute levels of JEB-G803* samples remained too low to be sufficiently analyzed (Figure 4C, left). When analyzed separately without NHKs, the increase in C17 signal was detectable (Figure 4C, right). Overall, G 0.003 μg/μL-based cocktail enhanced C17 restoration levels 1.7-fold compared to vehicle-treated JEB-G803* (Figure 4C, right), while absolute levels in JEB-G803* treated with the G-cocktail remained much lower than those observed in NHKs (Figure 4C, left).

PTC124-mediated readthrough in JEB-R145*

PTC124 did not affect the viability of JEB-R145* or NHKs (Figure 5A), nor did it impair *in vitro* wound closure (Figure 5B). While combination with CC-90009, NMDI-14, melatonin, and apocynin did not further enhance readthrough, PTC124 alone increased C17 protein levels by approximately 2-fold compared with vehicle-treated controls (Figure 5C). Restored C17 amount increased from 25.6% to 52% following PTC124 treatment in JEB-145* relative to NHKs (Figure 5D).

DISCUSSION

In our study, the exploratory TRID screening results suggested mutation-specific responses of JEB-C17 nonsense mutations to different readthrough compounds. PTC124 and/or G demonstrated readthrough efficacy among JEB-R145* and JEB-G803*, respectively. The JEB-R145* and JEB-G803* keratinocytes are compound heterozygous carrying a nonsense mutation together with a second variant. Therefore, the observed phenotype and treatment responses likely reflect the combined effects of both alleles. Although the

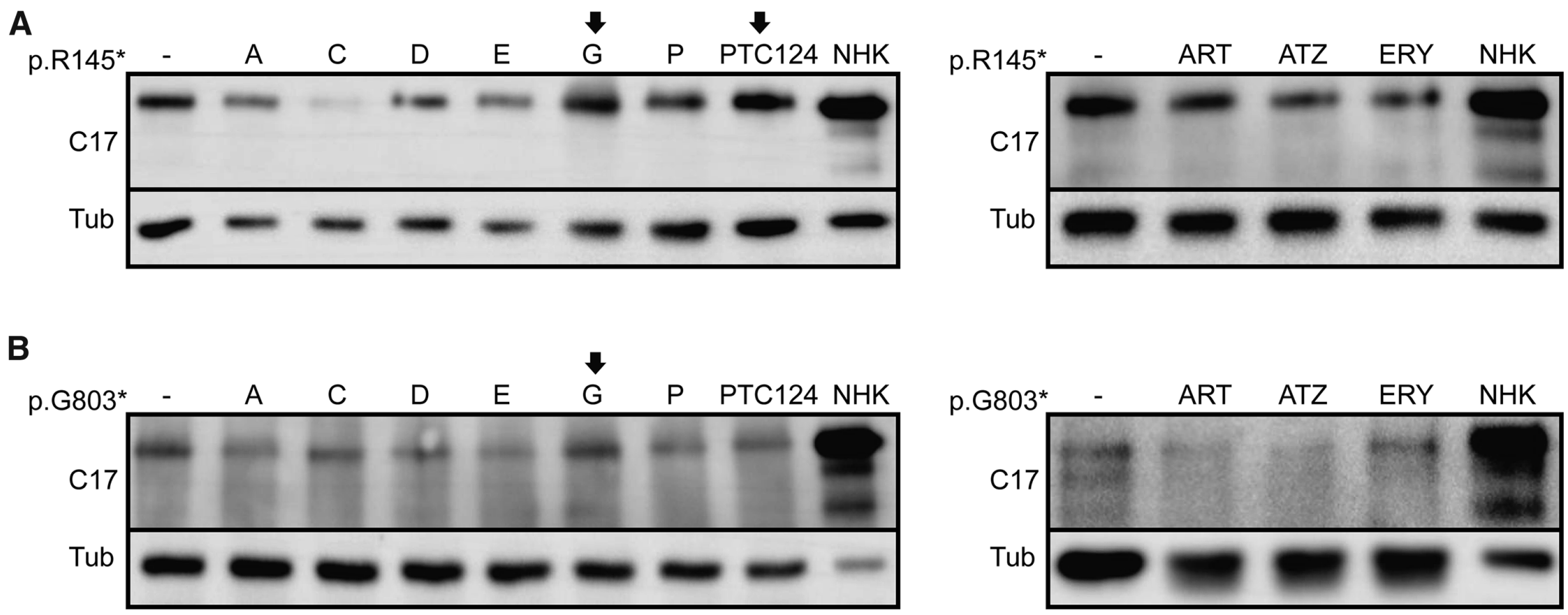

Figure 2. Screening of TRIDs in JEB-C17 keratinocytes

(A and B) Immunoblot analysis of C17 in JEB-R145* and -G803* following treatment with TRIDs (A 25 μM, ART 32 μM, ATZ 6.4 nM, C 2.7 μM, D 100 μM, E 3.5 μM, ERY 8 μM, G 0.25 or 1 μg/μL, P 1 μg/μL, and PTC124 35 μM) or vehicle control. Cells were treated for two consecutive days. Equal protein amounts were loaded for JEB-R145*, whereas 4-fold higher input was used for JEB-G803*. Tubulin served as a loading control. NHKs were used as reference controls; vehicle treated cells are indicated by "–". Data represent $n = 2$. A, amlexanox; ART, artesunate; ATZ, atazanavir; C, clitocine; D, 2,6-diaminopurine; E, ELX-02; ERY, erythromycin; G, gentamicin; P, paromomycin; PTC124, ataluren.

readthrough compounds evaluated in this study were selected to target PTCs, the potential contribution of the second allele to the therapeutic response cannot be excluded. Amlexanox, artesunate, atazanavir, clitocine, 2,6-diaminopurine, ELX-02, and erythromycin, did not significantly increase protein levels in any of the JEB-C17 keratinocytes. Variability in readthrough responses among different nonsense mutations within the same disease has been attributed to the PTC, as demonstrated using dual-reporter assays, with the hierarchy UGA>UAG>UAA, as well as to the sequence context surrounding the PTC with −1 U>G>C>A, and +4C > U>A>G.[34–36] However, in the present study, all JEB-C17 keratinocytes contained UGA as PTC and the no consistent sequence-context pattern was observed (Table 2).

Our results showed distinct NMD activity in different JEB-C17 nonsense mutations. We explored the NOG as a new enhancer of TRIDs readthrough activity. NOG potentiated both *COL17A1* mRNA level and C17 amount presence with aminoglycoside and CC-90009 in JEB-C17; nevertheless, different nonsense mutations p.W464*, p.G803*, and p.R145* elicited different responses to this drug. NOG was only effective in the presence of CC-90009, G and/or P in JEB-W464* and -G803*, while its combination with PTC124 did not induce readthrough activity in JEB-R145*. Moreover, aminoglycosides unresponsive nonsense mutation, p.R1226*, did not benefit from the addition of NOG or/and CC-90009. This indicates TRID selection is an initiative step for effective JEB-C17 readthrough therapy.

Given that JEB-R145* and -G803* responded to high dosage of G, we further evaluated G-based cocktail, which has anti-inflammatory properties.[15] Combination of low dosage of G with the cocktail consisted with NMD inhibitor (CC-90009, NMDI-14) and antioxidants (apocynin and melatonin) enhanced restored C17 without cell toxicity in JEB-G803*. But JEB-R145* did not respond to G-based cocktail. However, JEB-R145* showed robust answer to PTC124 while combination of PTC124 with the cocktail did not enhance C17 amount. Combining PTC124 with the cocktail did not further increase C17 amount compared with vehicle-treated control. The underlying mechanism remains unclear. However, this observation raises the possibility that combining different readthrough compounds may not necessarily result in additive or synergistic effects. Pharmacodynamic interactions may influence readthrough efficiency and/or translational dynamics.

Notably, PTC124 has successfully progressed through phase 3 clinical trials (ClinicalTrials.gov ID NCT01826487) in Duchenne muscular dystrophy.[37] Given that genotype-phenotype correlation studies have demonstrated that even 2%–4% of partially functional C17 can be biologically meaningful and may result in milder disease phenotypes,[32] G-based cocktail and PTC124 could be developed as potential readthrough treatment for JEB-G803* and JEB-R145*.

Our results revealed substantial variability in readthrough efficacy across different mutations and highlighted the molecular complexity underlying this phenomenon. It also indicated that the testing of TRIDs and determining the NMD status for nonsense mutations in patient derived cells should be considered a critical step in the development and optimization of therapeutic strategies. All together, these findings support a personalized approach to readthrough therapy for JEB-C17.

MATERIALS AND METHODS

Cell isolation and culture

Primary keratinocytes were isolated from skin biopsies obtained from patients with JEB-C17 carrying nonsense mutations and from healthy donors. The study was approved by the Ethics

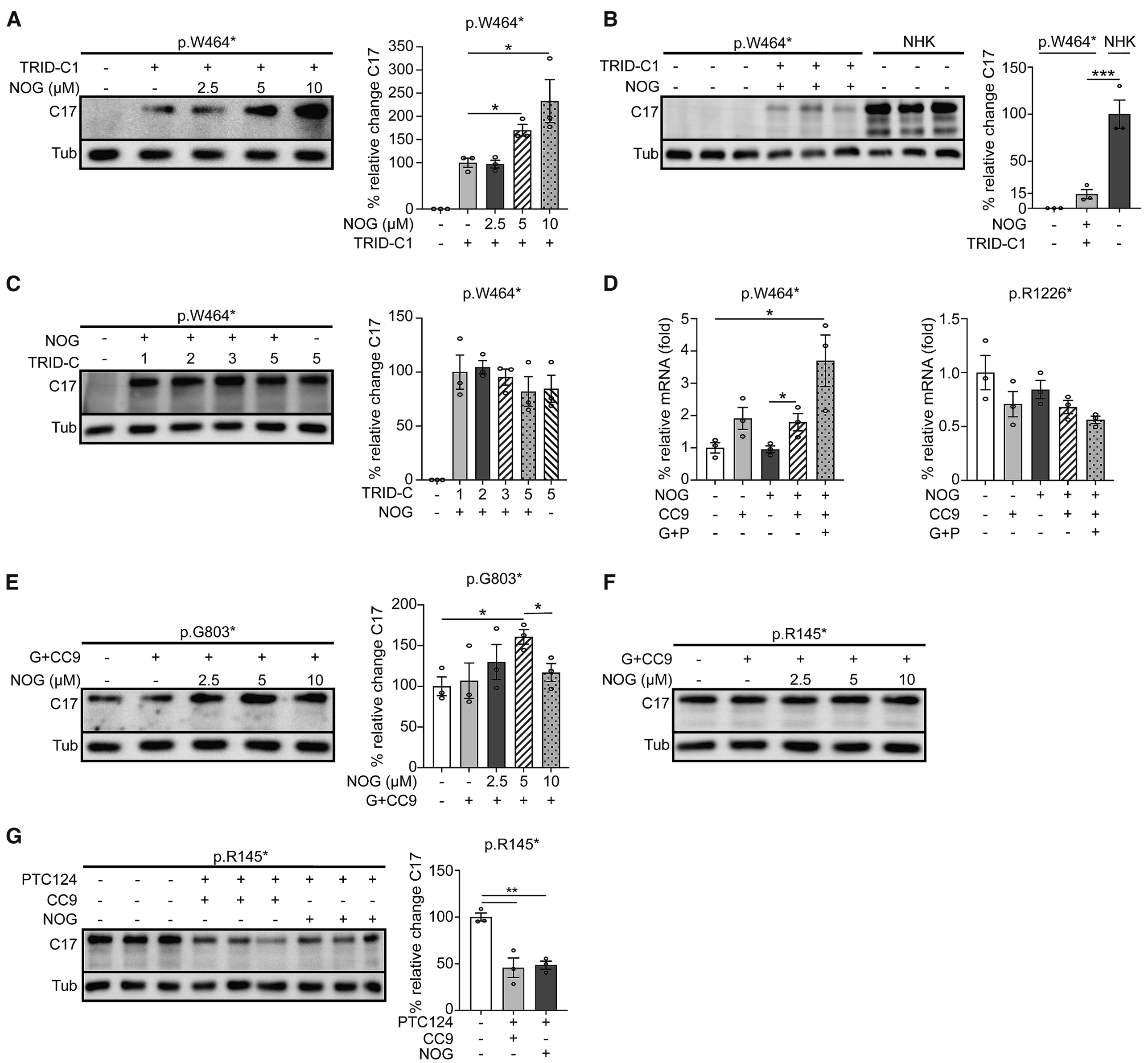

Figure 3. NOG enhances aminoglycoside-mediated readthrough

(A) JEB-W464* were treated with NOG 2.5–10 μM in combination with G 0.003 μg/μL, P 0.05 μg/μL, and CC-90009 30 nM. C17 levels were analyzed by immunoblotting and quantified. (B) Immunoblot and quantification of C17 in JEB-W464* keratinocytes treated with NOG in combination with TRID-C1 or with vehicle (–), relative to NHKs. (C) Immunoblot and quantification of C17 in JEB-W464* lysates. Cells were treated with NOG in different combinations: TRID-C1: G 0.003 μg/μL + P 0.05 μg/μL + CC-90009 30 nM, TRID-C2: TRID-C1 + melatonin 25 μM, TRID-C3: TRID-C1 + melatonin 25 μM + apocynin 25 μM, TRID-C5: TRID-C1 + melatonin 25 μM + apocynin 25 μM + NMDI-14 100 nM. (D) *COL17A1* mRNA expression in JEB-W464* and JEB-R1226* following treatment with NOG 5 μM, CC-90009 30 nM, G 0.003 μg/μL, P 0.05 μg/μL. (E–G) Immunoblots and quantifications of C17 in JEB-G803* and JEB-R145*. Cells were treated with NOG in combination of G 0.003 μg/μL and CC-90009 30 nM respectively or with PTC124 35 μM (G). Data are presented as mean ± SEM (*$p < 0.05$, **$p < 0.01$, ***$p < 0.001$; $n = 3$). Vehicle treated cells are indicated by "–". CC9, CC-90009.

Committee of the University of Freiburg (EK Freiburg 22–1208-S1). Cells were immortalized using E6/E7 genes (provided by Dr. F. Larcher, Madrid, Spain) and cultured in keratinocyte growth medium (PromoCell, Germany) at 37°C in a humidified atmosphere with 5% CO_2. Medium was changed every 2 days. Unless otherwise indicated, cells were treated one day after confluence with the specified compounds or vehicle control (DMSO) and harvested at 48 h after treatment.

Drug treatments

The following compounds were used at the indicated final concentrations: amlexanox (25 μM; Sigma-Aldrich), artesunate

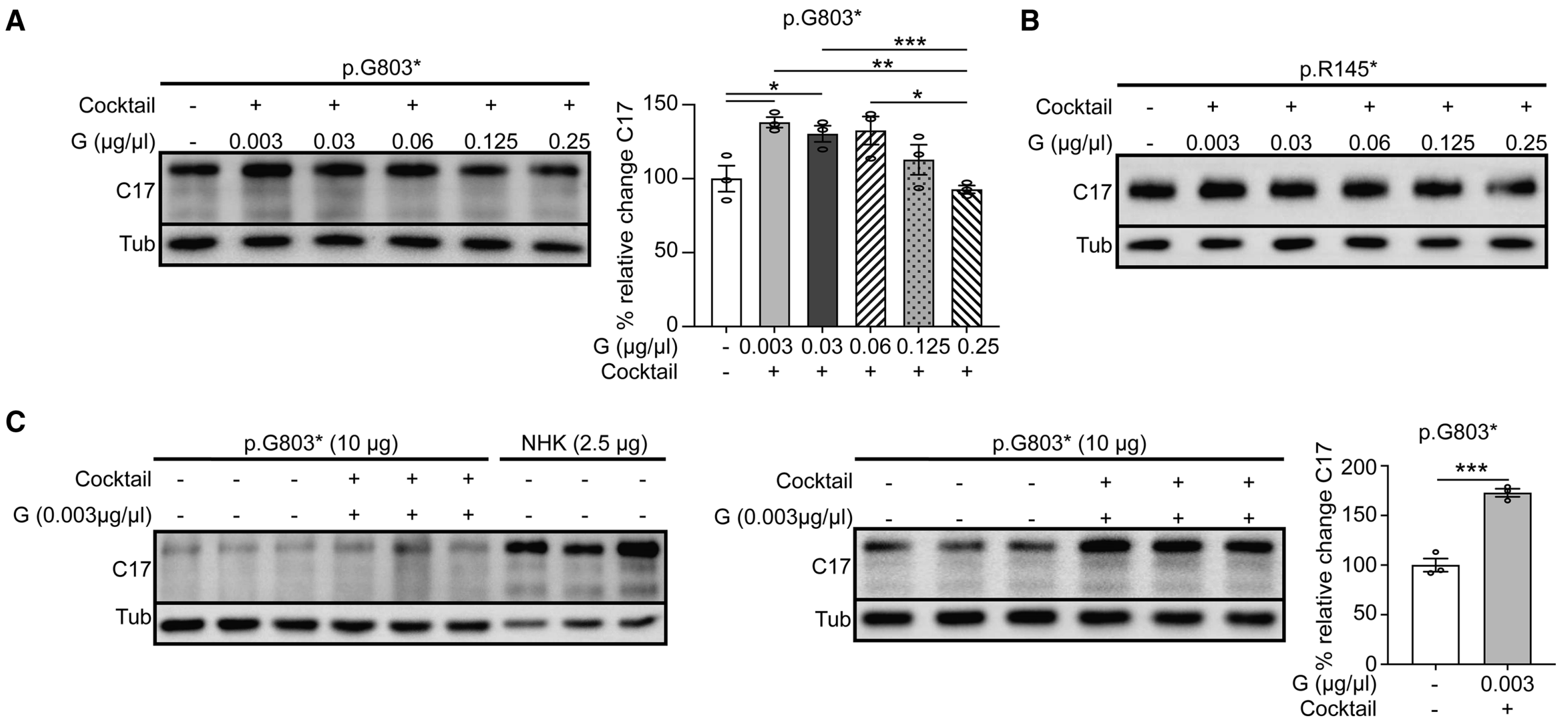

Figure 4. G-based TRID-cocktails

(A and B) JEB-R145* and -G803* were treated with vary G dosage (0.003, 0.03, 0.06, 0.125, 0.25 μg/μL) combined with CC-90009 30 nM, NMDI-14 100 nM, melatonin 25 μM, and apocynin 25 μM as indicated cocktail. C17 levels were analyzed by immunoblotting. (C) Immunoblot analysis of G 0.003 μg/μL-cocktail treated and vehicle treated JEB-G803* compared with NHKs. 4-fold higher protein input was used for JEB-G803* samples. Data are presented as mean ± SEM (*$p < 0.05$, **$p < 0.01$, ***$p < 0.001$; $n = 3$). Vehicle treated cells are indicated by "–".

(32 μM; Sigma-Aldrich), PTC124 (35 μM; Hycultec), atazanavir (6.4 nM; Sigma-Aldrich), clitocine (2.7 μM; Hycultec), 2,6-diaminopurine (100 μM; Sigma-Aldrich), ELX-02 (3.5 μM; Hycultec), erythromycin (8 μM; Sigma-Aldrich), G (0.003 to 1 μg/μL; Sigma-Aldrich), and P (0.05–1 μg/μL; Sigma-Aldrich). DMSO was used as vehicle control, and its final concentration did not exceed 0.1% in any condition.

Protein extraction and immunoblot analysis

Cells were seeded at a density of 5,000 cells/cm^2 in 6-well plates and cultured to one day after confluence prior to treatment. Total protein was extracted using a Triton X-100-containing lysis buffer. Protein samples were separated by 7% SDS-PAGE and transferred onto polyvinylidene fluoride (PVDF) membranes (Merck). Membranes were blocked with 5% BSA for 1 h at room temperature and incubated with primary antibodies against C17 (Abcam) overnight at 4°C or α-tubulin (Abcam) for 1 h at room temperature. After washing, membranes were incubated with horseradish peroxidase-conjugated secondary antibody (Santa Cruz Biotechnology; 1:10,000) for 60–90 min at room temperature. Signals were detected using enhanced chemiluminescence (ECL; GE Healthcare).

MTT assay

Keratinocytes were seeded in 96-well plates at a density of 2×10^4 cells per well and incubated for 24 h. Cells were then treated with the indicated compounds for 48 h. MTT reagent (Abcam) was added and incubated for 3 h at 37°C, followed by addition of solubilization solution according to the manufacturer's instructions (Abcam). Absorbance was measured at 590 nm using a microplate reader (Tecan). Cell viability was calculated relative to vehicle treated controls.

In vitro wound closure assay

Cells were seeded into two-well culture inserts (Ibidi, Germany) at a density of 2×10^4 cells/cm^2 and cultured for 24 h. Cells were treated with the indicated compounds or vehicle for 24 h prior to removal of inserts to create a defined gap. Images were acquired at 0 h and 24 h using a phase-contrast microscopy.

RNA isolation

Keratinocytes were seeded at 5,000 cells/cm^2 and cultured to confluence. One day after reaching confluence, cells were treated with the indicated compounds or vehicle control for 48 h. Total RNA was isolated using the RNeasy Plus Mini Kit (QIAGEN), and RNA concentration and purity were determined using a NanoDrop spectrophotometer. RNA samples were used for quantitative real-time PCR analysis. RNA sequencing (RNA-seq) analysis was performed on untreated cells by Novogene.

cDNA synthesis and quantitative real-time PCR

cDNA was synthesized using the RevertAid First Strand cDNA Synthesis Kit (Thermo Fisher Scientific). Quantitative real-time PCR was performed using SYBR Green Master Mix (Roche) on a QuantStudio 5 system (Applied Biosystems) according to the manufacturer's instructions. Gene-specific primers were used to amplify *COL17A1*, with TATA-box binding protein (TBP) serving as the endogenous reference gene for normalization. Reactions

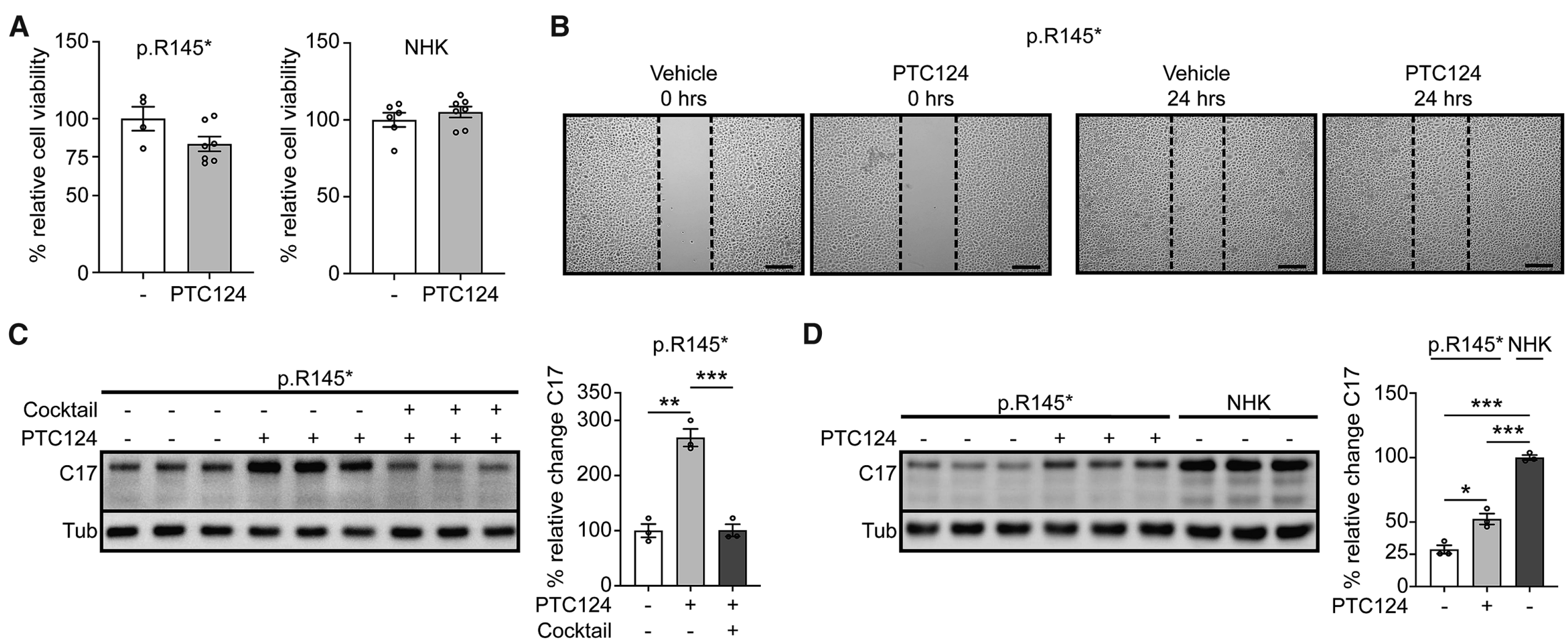

Figure 5. PTC124 mediated readthrough in JEB-R145*

(A) Cell viability of JEB-R145* and NHKs following treatment with PTC124 35 μM, assessed by (3-[4,5-dimethylthiazol-2-yl]-2,5 diphenyl tetrazolium bromide) (MTT) assay. (B) *In vitro* wound closure assay of JEB-R145* and NHKs after PTC124 treatment. Scale bars, 50 μm. (C) Immunoblot analysis of C17 in JEB-R145* treated with PTC124 alone or in combination with CC-90009 30 nM, NMDI-14 100 nM, melatonin 25 μM, and apocynin 25 μM. (D) Immunoblot and quantification of C17 in JEB-R145* cells treated with PTC124 or vehicle, with expression levels normalized to NHKs. Data are presented as mean ± SEM (*$p < 0.05$, **$p < 0.01$, ***$p < 0.001$; $n = 3$). Vehicle treated cells are indicated by "–".

were performed in two technical replicates and three biological replicates using a QuantStudio 5 Real-Time PCR System (Applied Biosystems, Thermo Fisher Scientific, USA). Amplification specificity was confirmed by melting curve analysis performed using QuantStudio Design and Analysis Software (Applied Biosystems). Relative gene expression was determined using the $2^{-\Delta\Delta Ct}$ method as previously described, with normalization to TBP expression.[38] Each experiment included three biological replicates and two technical replicates. Primer sequences are listed in Table S1.

Statistical analysis

All experiments were performed with at least three independent biological replicates unless otherwise indicated. Data are presented as mean ± standard error of the mean (SEM). Statistical analyses were performed using GraphPad Prism. Differences among multiple groups were analyzed by one-way ANOVA. Comparisons between two groups were performed using Student's *t* test. A *p* value <0.05 was considered statistically significant.

DATA AND CODE AVAILABILITY

The datasets analyzed in the present study are available from the corresponding author upon reasonable request.

ACKNOWLEDGMENTS

We thank all patients for their contribution. The study received approval from the Ethics Committee of the University of Freiburg (EK Freiburg 22-1208-S1). The project is funded by German Research Council (DFG HA 5663/3-1, FR 2524/5-1). Y.T. is supported by the State Scholarship Fund from China Scholarship Council (no. 202306210056). H.E.M. is funded by the EADV2024 Research Fellowship (no. RFE-2024-0007). Graphical abstract was created by using BioRender.com.

AUTHOR CONTRIBUTIONS

S.B.S. conceptualized the study; S.B.S. and C.H. curated the data; formal data analysis was performed by S.B.S., C.H., Y.T., H.E.M., and S.M.; C.H. acquired funding and provided resources; the investigation was carried out by Y.T., H.E.M., S.M., R.Z., and

Table 2. PTC identity and the surrounding sequences

				Nucleotide position						
cDNA level	Protein level	Exon	PTC	−3	−2	−1	+1	+2	+3	+4
c.433C>T	p.R145*	8	UGA	G	U	U	C	U	G	C
c.505C>T	p.R169*	9	UGA	A	G	U	U	C	G	G
c.1392G>A	p.W464*	17	UGA	A	G	C	U	G	G	A
c.2062C>T	p.R688*	26	UGA	C	U	C	G	G	U	G
c.2407G>T	p.G803*	34	UGA	C	C	A	G	C	U	C
c.3676C>T	p.R1226*	51	UGA	C	C	U	G	G	G	C

S.B.S.; S.B.S. and Y.T. developed the methodology; S.B.S. and C.H. administered the project and SBS supervised the study; validation was performed by Y.T., S.B.S., C.H., H.E.M., S.M., and R.Z.; S.B.S. prepared the original draft of the manuscript, and C.H., S.B.S., and Y.T. reviewed and edited the manuscript.

DECLARATION OF INTERESTS

The authors declare no competing interests.

SUPPLEMENTAL INFORMATION

Supplemental information can be found online at https://doi.org/10.1016/j.omtn.2026.103088.

REFERENCES

1. Has, C., Bauer, J.W., Bodemer, C., Bolling, M.C., Bruckner-Tuderman, L., Diem, A., Fine, J.-D., Heagerty, A., Hovnanian, A., Marinkovich, M.P., et al. (2020). Consensus reclassification of inherited epidermolysis bullosa and other disorders with skin fragility. Br. J. Dermatol. *183*, 614–627. https://doi.org/10.1111/bjd.18921.

2. Danescu, S., Negrutiu, M., and Has, C. (2024). Treatment of Epidermolysis Bullosa and Future Directions: A Review. Dermatol. Ther. *14*, 2059–2075. https://doi.org/10.1007/s13555-024-01227-8.

3. Hou, P.-C., Wang, H.-T., Abhee, S., Tu, W.-T., McGrath, J.A., and Hsu, C.-K. (2021). Investigational Treatments for Epidermolysis Bullosa. Am. J. Clin. Dermatol. *22*, 801–817. https://doi.org/10.1007/s40257-021-00626-3.

4. Hierl, M., Bischof, J., Liemberger, B., Kocher, T., Hainzl, S., Gruner, S., Trattner, L., Reichl, V., Klausegger, A., Aussel, C., et al. (2026). Prime editing as a promising therapeutic strategy for junctional epidermolysis bullosa. Mol. Ther. *34*, 817–831. https://doi.org/10.1016/j.ymthe.2025.10.054.

5. Petković, I., Bischof, J., Kocher, T., March, O.P., Liemberger, B., Hainzl, S., Strunk, D., Raninger, A.M., Binder, H.-M., Reichelt, J., et al. (2022). COL17A1 editing via homology-directed repair in junctional epidermolysis bullosa. Front. Med. *9*, 976604. https://doi.org/10.3389/fmed.2022.976604.

6. Condrat, I., He, Y., Cosgarea, R., and Has, C. (2018). Junctional Epidermolysis Bullosa: Allelic Heterogeneity and Mutation Stratification for Precision Medicine. Front. Med. *5*, 363. https://doi.org/10.3389/fmed.2018.00363.

7. Has, C., Sayar, S.B., Zheng, S., Chacón-Solano, E., Condrat, I., Yadav, A., Roberge, M., and Larcher Laguzzi, F. (2022). Read-Through for Nonsense Mutations in Type XVII Collagen−Deficient Junctional Epidermolysis Bullosa. J. Invest. Dermatol. *142*, 1227–1230.e4. https://doi.org/10.1016/j.jid.2021.09.018.

8. Carrard, J., and Lejeune, F. (2023). Nonsense-mediated mRNA decay, a simplified view of a complex mechanism. BMB Rep. *56*, 625–632. https://doi.org/10.5483/BMBRep.2023-0190.

9. Kurosaki, T., Popp, M.W., and Maquat, L.E. (2019). Quality and quantity control of gene expression by nonsense-mediated mRNA decay. Nat. Rev. Mol. Cell Biol. *20*, 406–420. https://doi.org/10.1038/s41580-019-0126-2.

10. Pan, H., Wu, X., Hu, B., Xiong, H., Luo, Y., and Zhou, J.-K. (2025). Nonsense-mediated mRNA decay: a key regulatory system engaged in cancer. Cell Commun. Signal. *23*, 489. https://doi.org/10.1186/s12964-025-02503-6.

11. Miao, K.L., Huynh, R., Woodley, D., and Chen, M. (2025). Therapeutic Opportunities in Overcoming Premature Termination Codons in Epidermolysis Bullosa via Translational Readthrough. Cells *14*, 1215. https://doi.org/10.3390/cells14151215.

12. Morais, P., Adachi, H., and Yu, Y.-T. (2020). Suppression of Nonsense Mutations by New Emerging Technologies. IJMS *21*, 4394. https://doi.org/10.3390/ijms21124394.

13. Zandanell, J., Wießner, M., Bauer, J.W., and Wagner, R.N. (2024). Stop codon readthrough as a treatment option for epidermolysis bullosa—Where we are and where we are going. Exp. Dermatol. *33*, e15042. https://doi.org/10.1111/exd.15042.

14. Yu, J., Tang, B., He, X., Zou, P., Zeng, Z., and Xiao, R. (2022). Nonsense Suppression Therapy: An Emerging Treatment for Hereditary Skin Diseases. Acta Derm. Venereol. *102*, adv00658. https://doi.org/10.2340/actadv.v102.353.

15. Sayar, S.B., and Has, C. (2024). Strategy for the Optimization of Read-Through Therapy for Junctional Epidermolysis Bullosa with COL17A1 Nonsense Mutation. J. Invest. Dermatol. *144*, 2221–2229.e1. https://doi.org/10.1016/j.jid.2024.02.027.

16. Sprenger, A., Küttner, V., Biniossek, M.L., Gretzmeier, C., Boerries, M., Mack, C., Has, C., Bruckner-Tuderman, L., and Dengjel, J. (2010). Comparative quantitation of proteome alterations induced by aging or immortalization in primary human fibroblasts and keratinocytes for clinical applications. Mol. Biosyst. *6*, 1579–1582. https://doi.org/10.1039/C003962D.

17. Atanasova, V.S., Jiang, Q., Prisco, M., Gruber, C., Piñón Hofbauer, J., Chen, M., Has, C., Bruckner-Tuderman, L., McGrath, J.A., Uitto, J., and South, A.P. (2017). Amlexanox Enhances Premature Termination Codon Read-Through in COL7A1 and Expression of Full Length Type VII Collagen: Potential Therapy for Recessive Dystrophic Epidermolysis Bullosa. J. Invest. Dermatol. *137*, 1842–1849. https://doi.org/10.1016/j.jid.2017.05.011.

18. Orlowski, G.M., Amano, S.U., Flanagan, K.E., Rieger, K.E., Marinkovich, M.P., and Wiss, K. (2023). Treatment With Ataluren for Wound Healing and Health Complications in a Patient With Junctional Epidermolysis Bullosa. JAMA Dermatol. *159*, 1145–1147. https://doi.org/10.1001/jamadermatol.2023.2077.

19. Kwong, A., Cogan, J., Hou, Y., Antaya, R., Hao, M., Kim, G., Lincoln, V., Chen, Q., Woodley, D.T., and Chen, M. (2020). Gentamicin Induces Laminin 332 and Improves Wound Healing in Junctional Epidermolysis Bullosa Patients with Nonsense Mutations. Mol. Ther. *28*, 1327–1338. https://doi.org/10.1016/j.ymthe.2020.03.006.

20. Levian, B., Hou, Y., Tang, X., Bainvoll, L., Zheng, K., Badarinarayana, V., Aghamohammadzadeh, S., and Chen, M. (2024). Novel readthrough agent suppresses nonsense mutations and restores functional type VII collagen and laminin 332 in epidermolysis bullosa. Mol. Ther. Nucleic Acids *35*, 102334. https://doi.org/10.1016/j.omtn.2024.102334.

21. Lincoln, V., Cogan, J., Hou, Y., Hirsch, M., Hao, M., Alexeev, V., De Luca, M., De Rosa, L., Bauer, J.W., Woodley, D.T., and Chen, M. (2018). Gentamicin induces *LAMB3* nonsense mutation readthrough and restores functional laminin 332 in junctional epidermolysis bullosa. Proc. Natl. Acad. Sci. USA *115*, E6536–E6545. https://doi.org/10.1073/pnas.1803154115.

22. Li, Y., Shen, J., Liang, J., Zheng, L., Chen, F., Yao, Z., and Li, M. (2020). Gentamicin induces COL17A1 nonsense mutation readthrough in junctional epidermolysis bullosa. J. Dermatol. *47*, e82–e83. https://doi.org/10.1111/1346-8138.15230.

23. Anderson-Crannage, M., Cairo, M.S., and Liao, Y. (2024). Reading through the nonsense: Gentamicin and ELX-02 as rescuing therapies for epidermolysis bullosa. Mol. Ther. Nucleic Acids *35*, 102368. https://doi.org/10.1016/j.omtn.2024.102368.

24. Rathner, A., Rathner, P., Friedrich, A., Wießner, M., Kitzler, C.M., Schernthaner, J., Karl, T., Krauß, J., Lottspeich, F., Mewes, W., et al. (2021). Drug Development for Target Ribosomal Protein rpL35/uL29 for Repair of LAMB3R635X in Rare Skin Disease Epidermolysis Bullosa. Skin Pharmacol. Physiol. *34*, 167–182. https://doi.org/10.1159/000513260.

25. Sinz, S.M., Koller, U., Liemberger, B., Hainzl, S., Klausegger, A., Hagen, J.v., Müller, N., Mohr, T., Gerner, C., Temaj, G., et al. (2025). Treating severe junctional epidermolysis bullosa with artesunate. skinonline. *1*, e169584. https://doi.org/10.1553/skindeep.2025.169584.

26. Wimmer, B., Friedrich, A., Poeltner, K., Edobor, G., Mosshammer, C., Temaj, G., Rathner, A., Karl, T., Krauss, J., von Hagen, J., et al. (2024). En Route to Targeted Ribosome Editing to Replenish Skin Anchor Protein LAMB3 in Junctional Epidermolysis Bullosa. JID Innov. *4*, 100240. https://doi.org/10.1016/j.xjidi.2023.100240.

27. Trzaska, C., Amand, S., Bailly, C., Leroy, C., Marchand, V., Duvernois-Berthet, E., Saliou, J.-M., Benhabiles, H., Werkmeister, E., Chassat, T., et al. (2020). 2,6-Diaminopurine as a highly potent corrector of UGA nonsense mutations. Nat. Commun. *11*, 1509. https://doi.org/10.1038/s41467-020-15140-z.

28. Friesen, W.J., Trotta, C.R., Tomizawa, Y., Zhuo, J., Johnson, B., Sierra, J., Roy, B., Weetall, M., Hedrick, J., Sheedy, J., et al. (2017). The nucleoside analog clitocine is a potent and efficacious readthrough agent. RNA *23*, 567–577. https://doi.org/10.1261/rna.060236.116.

29. Sun, J.G., Ruan, F., Zeng, X.L., Xiang, J., Li, X., Wu, P., Fung, K.P., and Liu, F.Y. (2016). Clitocine potentiates TRAIL-mediated apoptosis in human colon cancer cells by promoting Mcl-1 degradation. Apoptosis *21*, 1144–1157. https://doi.org/10.1007/s10495-016-1273-y.

30. Feng, T., Yamamoto, A., Wilkins, S.E., Sokolova, E., Yates, L.A., Münzel, M., Singh, P., Hopkinson, R.J., Fischer, R., Cockman, M.E., et al. (2014). Optimal Translational

Termination Requires C4 Lysyl Hydroxylation of eRF1. Mol. Cell *53*, 645–654. https://doi.org/10.1016/j.molcel.2013.12.028.

31. Wittenstein, A., Caspi, M., Rippin, I., Elroy-Stein, O., Eldar-Finkelman, H., Thoms, S., and Rosin-Arbesfeld, R. (2023). Nonsense mutation suppression is enhanced by targeting different stages of the protein synthesis process. PLoS Biol. *21*, e3002355. https://doi.org/10.1371/journal.pbio.3002355.

32. Kiritsi, D., Kern, J.S., Schumann, H., Kohlhase, J., Has, C., and Bruckner-Tuderman, L. (2011). Molecular mechanisms of phenotypic variability in junctional epidermolysis bullosa. J. Med. Genet. *48*, 450–457. https://doi.org/10.1136/jmg.2010.086751.

33. Hoffmann, J., Casetti, F., Reimer, A., Leppert, J., Grüninger, G., and Has, C. (2019). A Silent COL17A1 Variant Alters Splicing and Causes Junctional Epidermolysis Bullosa. Acta Derm Venerol *99*, 460–461. https://doi.org/10.2340/00015555-3133.

34. Mangkalaphiban, K., Fu, L., Du, M., Thrasher, K., Keeling, K.M., Bedwell, D.M., and Jacobson, A. (2024). Extended stop codon context predicts nonsense codon readthrough efficiency in human cells. Nat. Commun. *15*, 2486. https://doi.org/10.1038/s41467-024-46703-z.

35. Schilff, M., Sargsyan, Y., Hofhuis, J., and Thoms, S. (2021). Stop Codon Context-Specific Induction of Translational Readthrough. Biomolecules *11*, 1006. https://doi.org/10.3390/biom11071006.

36. Wangen, J.R., and Green, R. (2020). Stop codon context influences genome-wide stimulation of termination codon readthrough by aminoglycosides. eLife *9*, e52611. https://doi.org/10.7554/eLife.52611.

37. McDonald, C.M., Campbell, C., Torricelli, R.E., Finkel, R.S., Flanigan, K.M., Goemans, N., Heydemann, P., Kaminska, A., Kirschner, J., Muntoni, F., et al. (2017). Ataluren in patients with nonsense mutation Duchenne muscular dystrophy (ACT DMD): a multicentre, randomised, double-blind, placebo-controlled, phase 3 trial. Lancet *390*, 1489–1498. https://doi.org/10.1016/S0140-6736(17)31611-2.

38. Livak, K.J., and Schmittgen, T.D. (2001). Analysis of Relative Gene Expression Data Using Real-Time Quantitative PCR and the $2^{-\Delta\Delta CT}$ Method. Methods *25*, 402–408. https://doi.org/10.1006/meth.2001.1262.

OMTN, Volume *37*

Supplemental information

Distinct responses of *COL17A1* nonsense variants to readthrough drugs and NOG as a novel enhancer in junctional epidermolysis bullosa

Yan Tan, Haifa El Mabrouk, Rui Zhang, Sato Magassa, Cristina Has, and Saliha Beyza Sayar

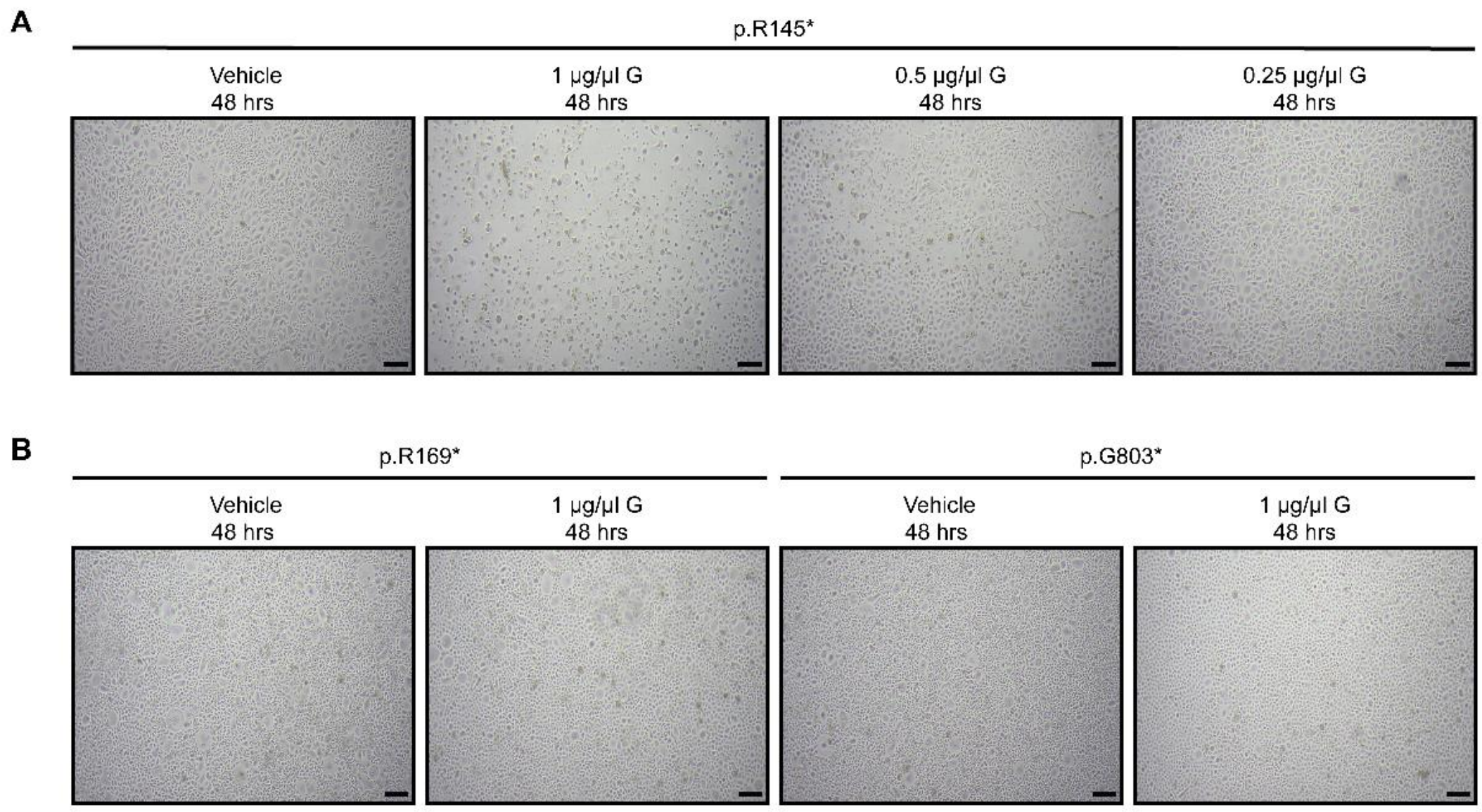

Figure S1. Morphology of JEB-C17 keratinocytes with high dosage G treatment.

(**A**) Morphological changes of JEB-R145* keratinocytes treated with G (1 µg/ µL, 0.5 µg/ µL, or 0.25 µg/ µL) respectively. (**B**) JEB-R169* and -G803* cells maintained normal morphology following treatment with G (1 µg/ µL). Cells were treated for two consecutive days and the pictures were taken at 48 hrs. Vehicle treated cells were used as reference controls. G, gentamicin. Bar = 50 µm.

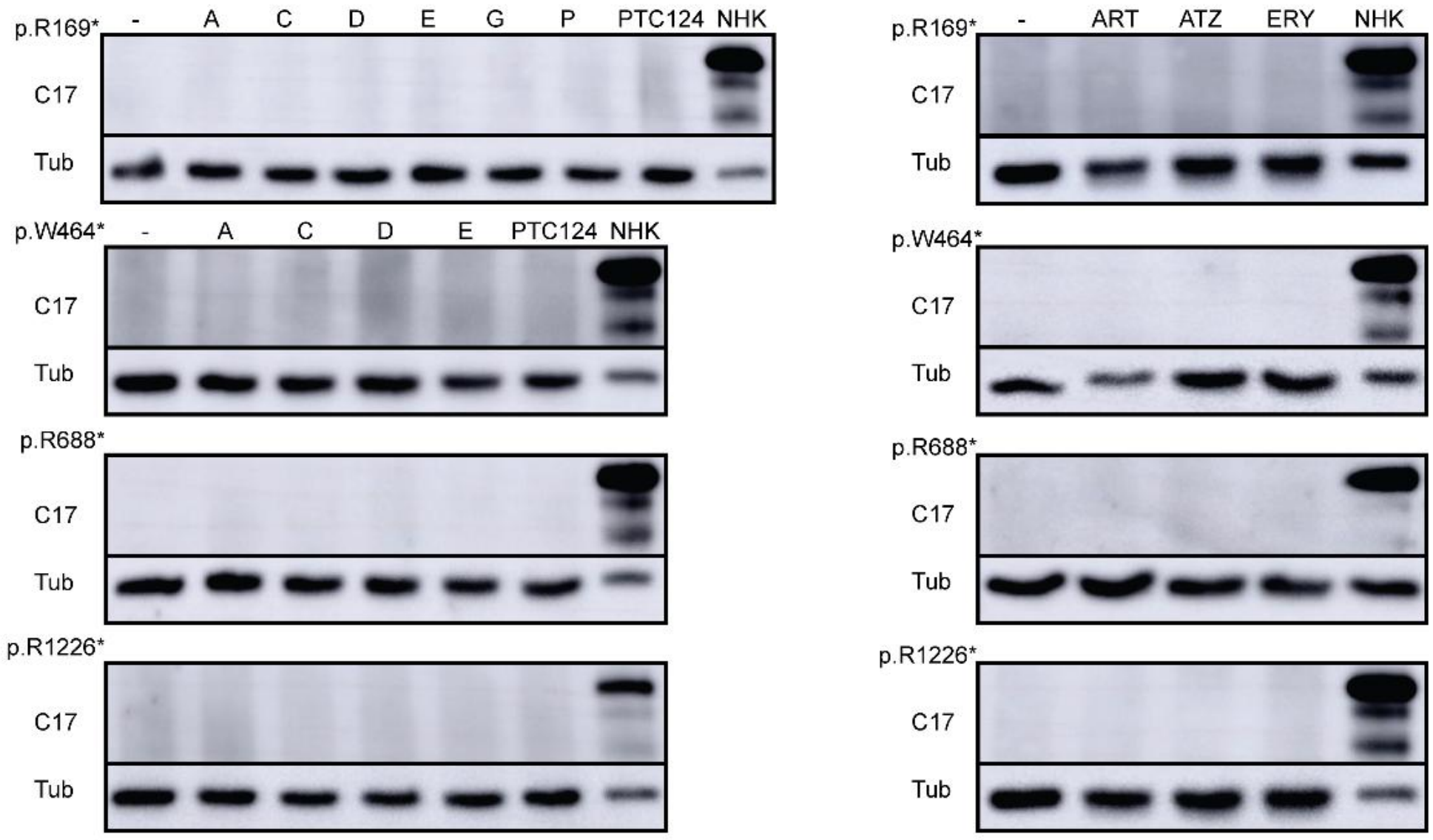

Figure S2. Screening of TRIDs in JEB-C17 keratinocytes.

Immunoblot analysis of C17 in JEB-R169*, -W464*, -R688* and -R1226* following treatment with TRIDs (A 25 µM, ART 32 µM, ATZ 6.4 nM, C 2.7 µM, DAP 100 µM, E 3.5 µM, ERY 8 µM, G 1µg/µL, P 1µg/µL, and PTC124 35 µM) versus vehicle. Cells were treated for two consecutive days. Fourfold higher input was used for JEB-C17 keratinocytes as compared to NHKs. Tubulin served as a loading control. NHKs were used as reference controls; vehicle treated cells are indicated by "–". Data represent n = 2. A, amlexanox; ART, artesunate; ATZ, atazanavir; C, clitocine; D, 2,6-diaminopurine; E, ELX-02; ERY, erythromycin; G, gentamicin; P, paromomycin; PTC124, ataluren.

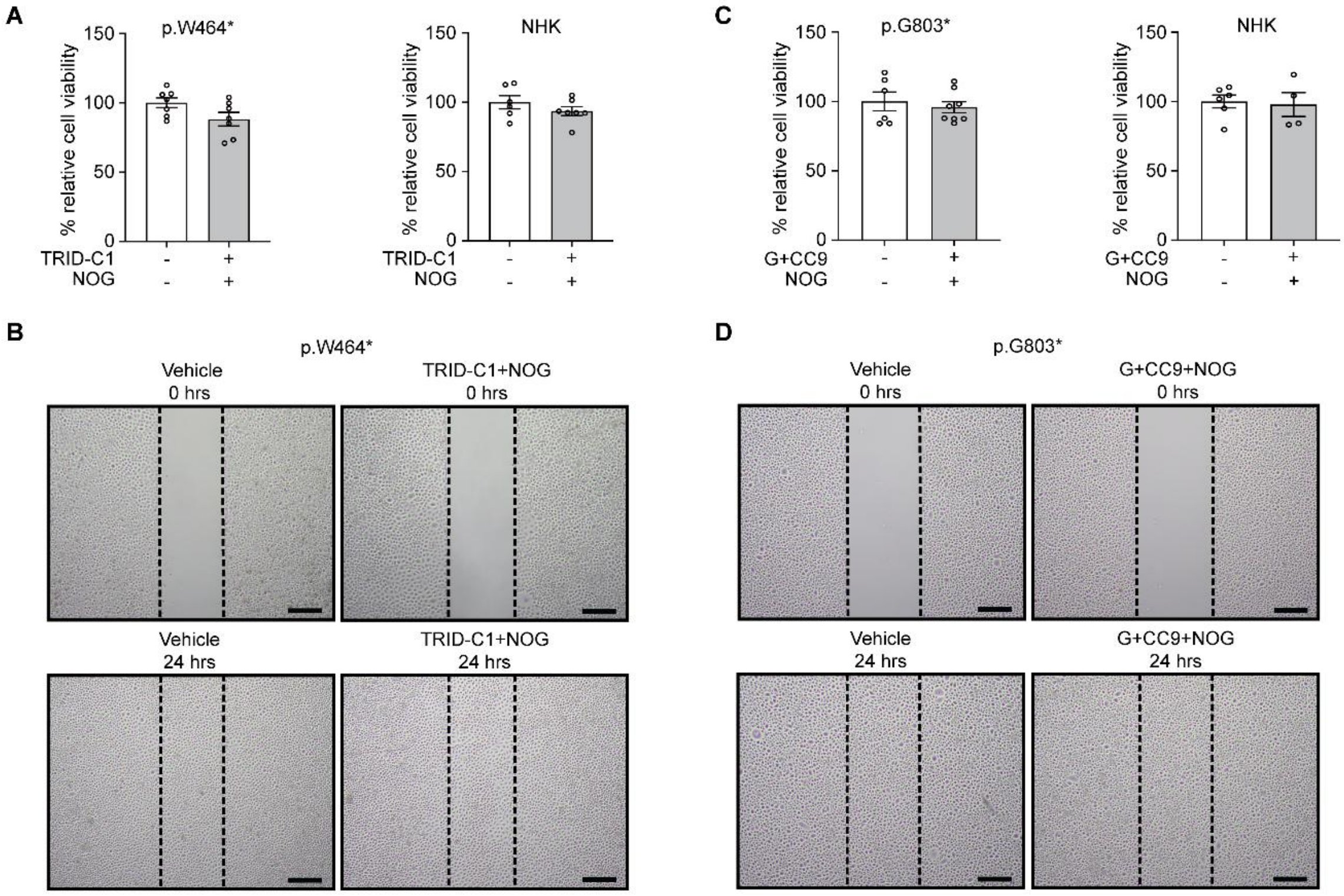

Figure S3. Evaluation of NOG effect on cell viability and *in vitro* wound closure.

(**A**) The graph depicts the relative change of cell viability of JEB-W464* and NHKs treated with NOG 5 µM in combination with TRID-C1 (G 0.003 µg/µL, P 0.05 µg/µL, and CC-90009 30 nM). (**B**) *in vitro* wound closure assay of JEB-W464* and NHKs treated with NOG 5 µM in combination with TRID-C1. (**C**) The graph shows the relative change of cell viability of JEB- JEB-G803* and NHKs treated with NOG 5 µM in combination with G 0.003 µg/µL and CC-90009 30 nM. (**D**) *in vitro* wound closure assay of JEB-G803* and NHKs treated with NOG 5 µM in combination with G 0.003 µg/µL and CC-90009 30 nM. Bars = 50 µm. Data are presented as mean ± SEM (**P* < 0.05, ***P* < 0.01, ****P* < 0.001; n = 3). Vehicle treated cells are indicated by "–". CC9, CC-90009.

Table S1. Primers used for RT-qPCR

Primer Name	Direction	Sequence 5′−3′	Annealing Temperature (°C)	Product Size (bp)
*COL17A1*_ex40-44	F	GAAACCTTCCTCTCCGGCC	60	232
*COL17A1*_ex40-44	R	CTAGGAATGCCAAGAGCCCC	60	232
18S	F	GTAACCCGTTGAACCCCATT	60	151
18S	R	CCATCCAATCGGTAGTAGCG	60	151
TBP	F	CATTGGACTAAGATAGGGATTCCG	60	93
TBP	R	ATGGATCAGAACAACAGCCTGC	60	93

Legend: F, forward; R, reverse.
